# Untargeted Metabolite Profiling of Adipose Tissue in Rats Exposed to Mepiquat

**DOI:** 10.3390/foods12040867

**Published:** 2023-02-17

**Authors:** Chuanqin Hu, Xinyu Song, Zhenzhen Shao, Yingli Liu, Jing Wang, Baoguo Sun

**Affiliations:** China-Canada Joint Lab of Food Nutrition and Health (Beijing), Beijing Advanced Innovation Center for Food Nutrition and Human Health, Beijing Engineering and Technology Research Center of Food Additives, Beijing Laboratory for Food Quality and Safety, Beijing Technology and Business University (BTBU), 11 Fucheng Road, Beijing 100048, China

**Keywords:** mass spectrometry, food safety, metabolomic, untargeted metabolite profiling, mepiquat

## Abstract

Mepiquat (Mep) is a contaminant produced by Maillard reaction with reducing sugar, free lysine and an alkylating agent under typical roasting conditions, particularly in the range of 200–240 °C. It has been reported that exposure to Mep is harmful to rats. However, its metabolic mechanism is still not clear. In this study, untargeted metabolomics was used to reveal the effect of Mep on the metabolic profile of adipose tissue in Sprague-Dawley rats. Twenty-six differential metabolites were screened out. Eight major perturbed metabolic pathways were found, which were linoleic acid metabolism, Phenylalanine, tyrosine, and tryptophan biosynthesis, phenylalanine metabolism, arachidonic acid metabolism, Glycine, serine, and threonine metabolism, glycerolipid metabolism, Alanine, aspartate, and glutamate metabolism, and glyoxylate and dicarboxylic acid metabolism. This study lays a solid foundation for clarifying the toxic mechanism of Mep.

## 1. Introduction

Mepiquat (N,N-dimethylpiperidinium, C_7_H_16_N, Mep) is a plant growth regulator usually used in agriculture with high efficiency. Maximum residue limits (MRLs) for mepiquat in foods have been established in many countries or regions [1]. It is also a contaminant produced by the Maillard reaction with reducing sugar, free lysine and an alkylating agent under typical roasting conditions, particularly in the range of 200–240 °C. The highest levels of Mep were found in roasted barley and soluble coffee, 640 μg/kg and 1400 μg/kg, respectively [2]. Levels of Mep were 1064 μg/kg and 293 μg/kg in potatoes and broccoli after roasting at 240 °C and 260 °C respectively for 20 min in the oven [3]. Mep levels in oven-cooked beef were up to 82.5 μg/kg after 10 min at 250 °C [4]. Therefore, Mep has become a potential factor affecting food safety. Mep formed in thermally processed foods may raise human exposure. The European Food Safety Authority (EFSA) has reported that exposure to Mep is harmful to rats. It was found that Mep can cause kidney vacuolization and liver and spleen damage [5], but its metabolic mechanism is still not clear.

Metabolomics can evaluate the global metabolic profiling of molecular responses in organisms that are disturbed by the outside world, and generate a large amount of metabolic pathway information for deciphering the metabolic networks altered by various stimuli [6]. Techniques such as nuclear magnetic resonance [7] and mass spectrometry [8,9] are used in metabolomic analysis. Nuclear magnetic resonance-based metabolomic analysis can achieve unbiased and high-throughput analysis of biological samples, but it has limited dynamic range, low sensitivity, and small metabolite detection coverage. As a result, its application is limited. Mass spectrometry has advantages of strong specificity, high resolution, and high sensitivity. For the analysis of biological samples, it can simultaneously detect hundreds of small molecule metabolites [10]. White adipose tissue is an important participant in energy regulation of body. It stores excess ingested fatty acids in the form of triglycerides and meets the energy needs of other organs by releasing fatty acids. In addition, it is an endocrine organ affecting essential metabolic processes, including the dynamic balance of lipid and glucose [11].

To date, there is no metabolomics study of adipose tissue of mepiquat-exposed rats. In this study, gas chromatography-mass spectrometry (GC-MS) was used to detect changes of metabolites in adipose samples. In addition, we examined histopathological changes of adipose tissues in rats. The method was established to find more differential metabolites to comprehensively reveal the mechanism of Mep toxicity.

## 2. Materials and Methods

### 2.1. Chemicals and Reagents

Mepiquat (purity > 98%) was purchased from Sigma-Aldrich (Buchs, Switzerland). Methanol was HPLC grade and obtained from Fisher (Fair Lawn, NJ, USA). All standard compounds and 4-chloro-DL-phenylalanine were gained from Sigma or Sigma-Aldrich (St. Louis, MO, USA), Sigma-Aldrich also provided the derivatization reagent (99% MSTFA + 1% TMCS, pyridine, and methoxyamine). All other chemicals were analytical grade.

### 2.2. Animals and Treatments

This experiment was carried out in the SPF (Specific Pathogen Free) animal laboratory of animal center of Peking University Health Science Center (Beijing, China). Conditions of the breeding environment were controlled as follows: 12 h dark/light cycle, temperature 22 °C ± 1 °C, and relative humidity 60 ± 5%. All experimental treatments were carried out according to the European Community guidelines for experimental animal use. The study plan was agreed by the Experimental Animal Protection and Use Committee of Peking University (Approval No. LA2019032). Thirty male Sprague-Dawley rats aged 5–6 weeks were randomly divided into normal diet group (Normal Diet, ND, *n* = 10), low-dose group (Low-dose Diet, LD, *n* = 10) and high-dose group (High-dose Diet, HD, *n* = 10). Rats were fed with standard laboratory feed and with ad libitum access to diet and water. One week later, the weight of rats was 200 ± 15 g. LD_50_ of mepiquat is 464 mg/kg bw [5]. Rats in LD group and HD group were given 15 mg/kg and 150 mg/kg Mep dissolved in distilled water by oral gavages once every morning [12]. Rats in ND group was given 10 mL/kg distilled water by the same way. Body weight were recorded daily. Rats were sacrificed by decapitation, adipose tissues were collected quickly, and some of the adipose tissues were frozen at −80 °C, while the rest were fixed in 10% neutral buffered formalin solution for histological analysis.

### 2.3. Sample Preparation

Fifty milligram white adipose tissue was homogenized in 2.0 mL of chloroform/methanol (*v*/*v*, 2:1). After centrifugation at 10,000 r/min for 5 min at 4 °C, supernatant was collected and the same procedure was used twice to extract the residue. The obtained complete supernatant was dried with nitrogen after being centrifuged for 5 min at 12,000 r/min. In total, 10 μL of 4-chloro-DL-phenylalanine (1.05 mg/mL in water) was added to each sample. Every sample was lyophilized and derivatized by adding 80 μL of MSTFA at 70 °C for 3 h. Every sample was mixed with 165 μL chloroform, vortexed, and centrifuged at 15,000 r/min for 15 min at 4 °C. Supernatant was transferred to GC-MS vials for analysis. Quality control (QC) samples were made by mixing the same volume (10 μL) of each sample. QC sample was run once every six samples during the assay [13].

### 2.4. GC-MS Analysis

Agilent 7890A/5975C gas chromatography mass spectrometer was used with an HP-5 MS capillary column (30 m × 250 μm i.d., 0.25 μm). The carrier gas was chromatographic grade helium, and the constant flow rate was 1.0 mL/min. The temperature program was set as follows: the initial temperature was 60 °C, held for 2 min, increased to 240 °C at a rate of 5 °C/min, then held for 3 min, and raised to 290 °C at a rate of 12 °C/min, maintained for 2 min. Injector temperature was set at 280 °C. Solvent delay was 5 min. Splitless injection mode was used. Injection volume was 1 μL. The mass spectrometry data were obtained in full scan mode (*m*/*z* 50 to 650). Electron energy was 70 eV. Identification of compounds was carried out by authentic standards and the NIST library (2014) (Gaithersburg, MD, USA) [14].

### 2.5. Data Processing and Multivariate Analysis

Data analysis was performed based on a previous research [14]. The normalized data were imported into SIMCA-P14.1 software (Umetrics, Umeå, Sweden). Data were analyzed with PCA model and PLS-DA model. Differential metabolites were determined based on variable importance in the projection (VIP > 1.0) and *p*-value (*p* < 0.05) gained from the Mann–Whitney U test in SPSS22.0 software (SPSS Inc., Chicago, IL, USA). Metaboanalyst 5.0 (https://www.metaboanalyst.ca/ (accessed on 26 February 2022)) and cytoscape software was utilized for metabolic pathway analysis.

### 2.6. Receiver Operator Characteristic (ROC) Analysis

The ROC analysis was used for assessing the diagnostic ability of the differential metabolites to classify rats into a low or high Mep exposure. The area under ROC curve (AUC) from 0.5 to 1.0 showed diagnostic accuracy from no discrimination to good classification. The ROC analysis was completed by SPSS 22.0 software.

## 3. Results and Discussion

### 3.1. Weight Change

In this study, weight gained in Mep exposure groups were slower than that in the normal diet group. It indicated that Mep significantly inhibited the weight gain of rats (*p* < 0.05), as shown in Figure S1.

### 3.2. Histopathological Analysis

**Figure 1:**
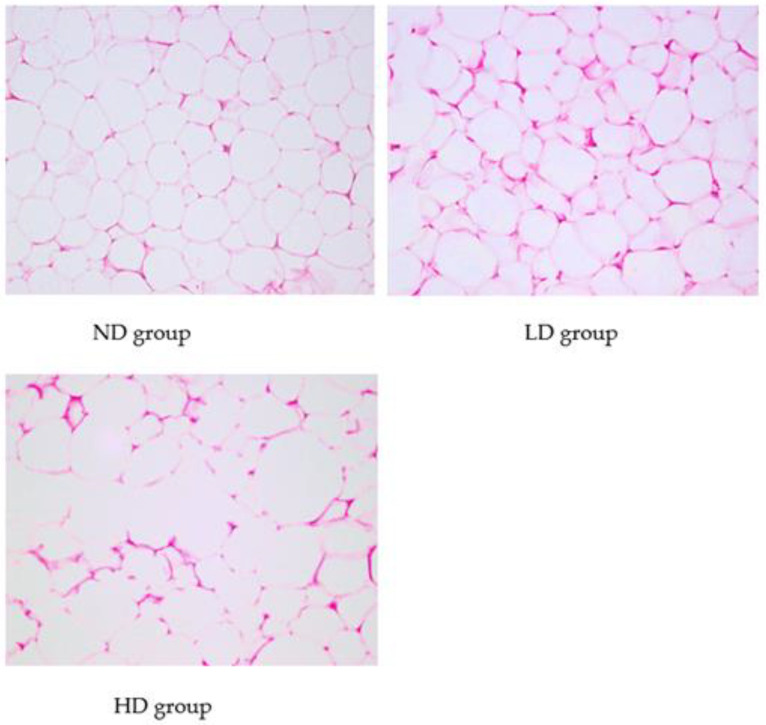
HE staining of adipose tissues from different groups (original magnification: 400×) (ND group: Normal Diet group, *n* = 10; LD group: Low-dose Diet group, *n* = 10; HD group: High-dose Diet group, *n* = 10).

As shown in Figure 1, adipose tissue cells in ND group were closely arranged, and were similar in size with clear outlines. It showed disordered cell arrangement, different sizes, and irregular changes in cell shape in LD group. The number of cells increased in the same field of view. In HD group, it was observed that cells size and shape changed greatly. Some cells in HD group increased in size by a factor of two or more compared to those in ND group. In HD group, and the outline of cells was deformed and blurred, and the arrangement was disordered, accompanied by inflammatory cell infiltration.

### 3.3. GC-MS Analysis of Adipose Tissue

Differential metabolites were screened based on the remarkable differences (VIP > 1.0 and *p* < 0.05) of HD/ND in abundance. A total of twenty-six differential metabolites in adipose were quantified by normalization to 4-chloro-DL-phenylalanine (internal standard). The relative levels of metabolites were shown as fold changes (Table 1).

Twenty-six metabolites in Table 1 were regarded as differential metabolites. Elevated concentrations of many metabolites were found in rats. Compared with ND group, the levels of amino acids, such as glycine, valine, leucine, isoleucine, serine, proline, phenylalanine, and alanine, were higher in HD group. By contrast, fatty acids including propanoic acid, hexadecanoic acid, octadecanoic acid, arachidonic acid, and oleic acid decreased in rats exposed to Mep. In comparison with ND group, contents of propionate, phosphoric acid, acetamide, creatinine, D-mannitol, pentanedioic acid, and acetic acid increased, while levels of lactic acid, urea, cholesterol, and glycerol decreased in HD group.

### 3.4. Adipose Tissue Metabolomics Analysis

Data of adipose samples from three groups were visualized. In the PCA model, it was found that the ND, LD, and HD groups was clearly separated. The PLS-DA model showed that scattered points of three groups were clearly distinguished too. Mep-exposed groups were significantly shifted from the ND group, and the HD group was more significantly shifted than the LD group. This showed that with the increase of administration doses, significant changes were found in adipose tissue. The validity of the model was confirmed by a permutation test and the cross-validation parameter Q^2^.

**Figure 2:**
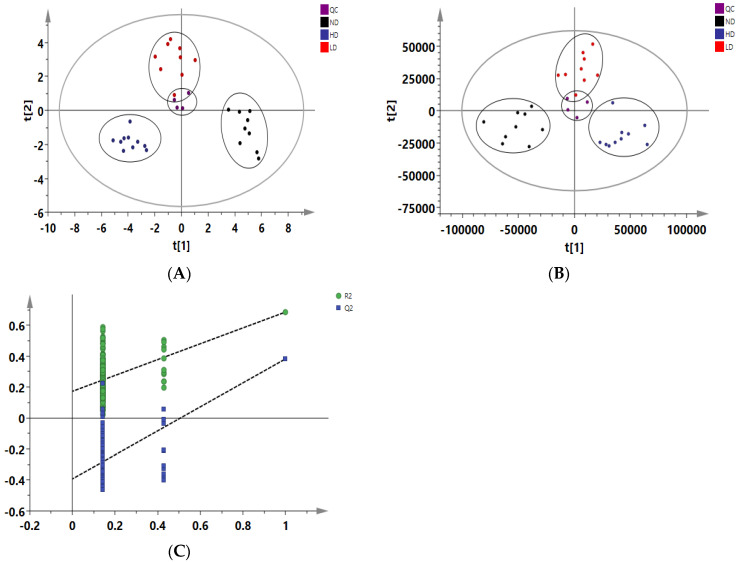
PCA and PLS-DA score plot derived from the GC-MS analysis of adipose tissue from the ND, LD, and HD groups (**A**) PCA score map (R^2^X = 77.7%, Q^2^ = 56.6%); (**B**) PLS-DA score map (R^2^X = 76.7%, R^2^Y = 84 %, Q^2^ = 73.3%); (**C**) Permutation experiment of PLS-DA model (n = 999) R^2^ = (0.0, 0.175), Q^2^ = (0.0, −0.403) (PCA: Principal Component Analysis; PLS-DA: Partial Least Squares Discrimination Analysis; ND group: Normal Diet group, *n* = 10; LD group: Low-dose Diet group, *n* = 10; HD group: High-dose Diet group, *n* = 10).

Analytical testing for stability and reproducibility was performed with quality control (QC) samples. In Figure 2, the QC samples have little change and the distribution was relatively concentrated. Its reproducibility was good, indicating that the system was stable and reliable. In Figure 2A, R^2^X (cum) = 77.7%, Q^2^ (cum) = 56.6%. In Figure 2B, R^2^X (cum) = 76.7%, R^2^Y (cum) = 84%, Q^2^ (cum) = 73.3%. It showed that the two models have good quality and predictive ability. By performing a permutation test, it was verified whether the PLS-DA model was overfitting to evaluate the reliability of the model. The permutation experiment (number of permutations n = 999), R^2^ = (0.0, 0.175) and Q^2^ = (0.0, −0.403), proved that the model was not over fitted.

**Figure 3:**
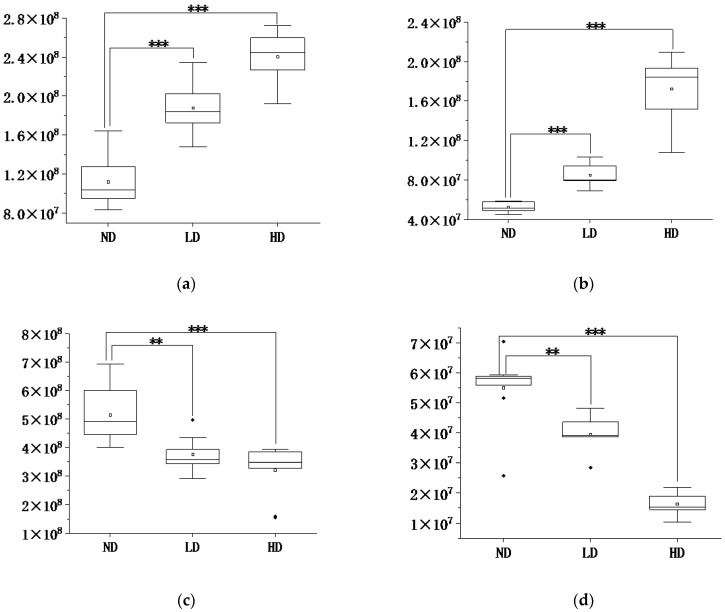
Boxplots of differential metabolites in adipose tissue of rats in HD, LD, and ND groups: (**a**) Glycine; (**b**) Isoleucine; (**c**) Octadecanoic acid; (**d**) Glutamine (“**” means *p* < 0.01, “***” means *p* < 0.001. ND group: Normal Diet group, *n* = 10; LD group: Low-dose Diet group, *n* = 10; HD group: High-dose Diet group, *n* = 10).

Boxplots were used for comparing changes in relative levels of metabolites in adipose tissue. In comparison with the ND group, contents of glycine (VIP = 1.07, *p* < 0.001) and isoleucine (VIP = 1.19, *p* < 0.001) increased in Mep-exposed groups; however, levels of octadecanoic acid (VIP = 1.79, *p* < 0.001) and glutamine (VIP = 1.01, *p* < 0.001) decreased in Mep-exposed groups (Figure 3). This indicated a dose-dependent relationship in groups with Mep exposure.

### 3.5. ROC Curve

Differential metabolites were subjected to ROC analysis. ROC analysis results with the MS quantitative data of twenty-six metabolites are shown in Table S1, of which metabolites exhibited a good diagnostic (AUC > 0.8). These differential metabolites can distinguish between normal diet rats and relatively high exposure rats with Mep exposure.

### 3.6. Metabolic Pathway Analysis

**Figure 4:**
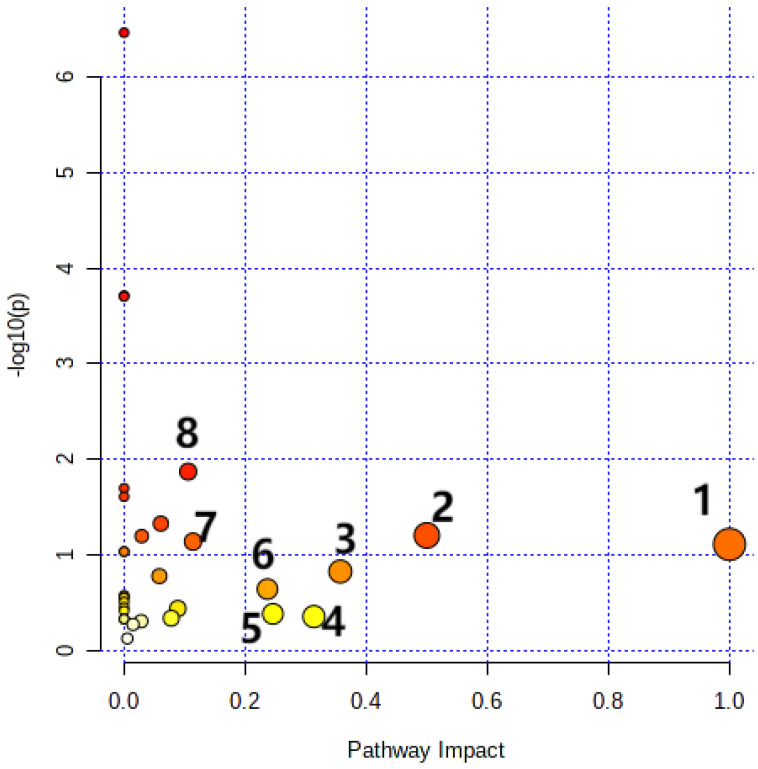
Disturbed metabolic pathways in adipose tissue of rats from three groups. (1) Linoleic acid metabolism; (2) Phenylalanine, tyrosine, and tryptophan biosynthesis; (3) Phenylalanine metabolism; (4) Arachidonic acid metabolism; (5) Glycine, serine, and threonine metabolism; (6) Glycerolipid metabolism; (7) Alanine, aspartate, and glutamate metabolism; (8) Glyoxylate and dicarboxylate metabolism.

Twenty-six differential metabolites are imported into MetaboAnalyst 5.0 for visualization. Pathway analysis was performed by the Rattus norvegicus (rat, 81 pathways) pathway library and the compound name of differential metabolites. The impact-value threshold obtained from pathway topology analysis was 0.10. Metabolic pathways with an impact value greater than 0.10 are linoleic acid metabolism, Phenylalanine, tyrosine, and tryptophan biosynthesis, phenylalanine metabolism, arachidonic acid metabolism, Glycine, serine, and threonine metabolism, glycerolipid metabolism, Alanine, aspartate, and glutamate metabolism, and glyoxylate and dicarboxylate metabolism. A summary of the pathway analysis was shown in Figure 4 and Table 2. Linoleic acid metabolism is an important metabolic pathway with the highest impact value (Figure S2).

**Figure 5:**
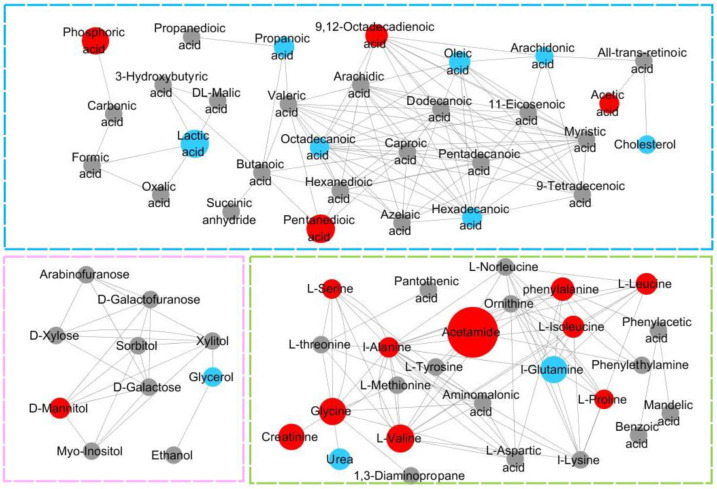
Metabolic pathways associated with Mep exposure when comparing with ND group. The depicted networks revealed red nodes represent significantly upregulated metabolites, blue nodes represent significantly downregulated metabolites that were involved in the affected metabolites, and gray nodes represent no significant changes in metabolites. The size of the node was positively correlated with the fold change between the HD group and ND group (Mep: Mepiquat; ND group: Normal Diet group, *n* = 10; LD group: Low-dose Diet group, *n* = 10; HD group: High-dose Diet group, *n* = 10).

**Figure 6:**
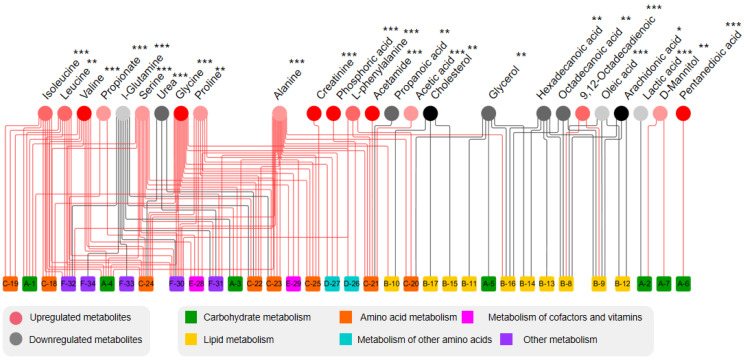
Network analysis of differential metabolites and metabolic pathways at Mep-exposed groups shows that there are twenty-six differential metabolites. “*” means *p* < 0.05, “**” means *p* < 0.001, “***” means *p* < 0.0001. The red circles and black circles show upregulation and downregulation of metabolites, respectively. Intensity of the color displays the metabolite fold change. A total of thirty-four metabolic pathways (squares) were classified as six metabolic pathways (Table S2), with different metabolites connected by the red line (upregulation) and black line (downregulation).

**Figure 7:**
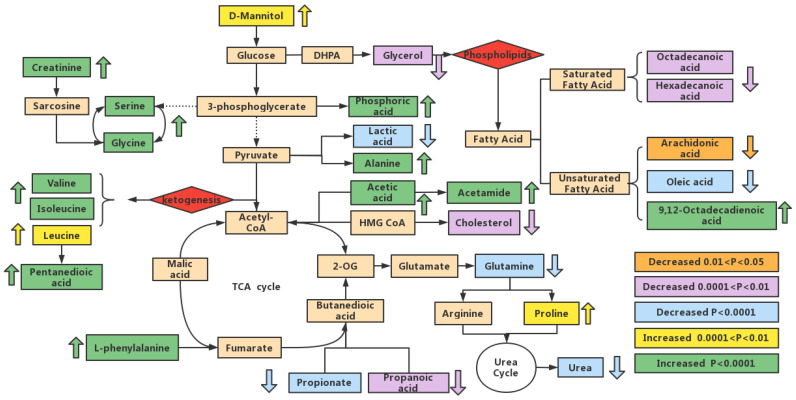
Potential metabolic pathways disturbed in rats after Mep exposure.

KEGG database were used for analyzing metabolic networks related to differential metabolites. Metabolic network was formed in MetaMapp with integrating biochemical networks and chemical relationships. Metabolomics datasets were efficiently visualized as network graphs in Cytoscape using MetaMapp. In Figure 5, levels of all carbohydrates and amino acids are upregulated. Among all the differential metabolites, acetamide has the largest change. The blue-, green-, and pink-dotted boxes represent fatty acid metabolism, amino acid metabolism, and carbohydrate metabolism, respectively. In Figure 6, there are more upregulated metabolic pathways in the Mep-exposed groups, especially carbohydrate metabolism and amino acid metabolism. For example, propionate metabolism, pyruvate metabolism, and valine, leucine, and isoleucine biosynthesis are enriched in various upregulated metabolites. In Mep-exposed groups, there are 16 upregulated and 10 downregulated differential metabolites. The key upregulated metabolite alanine participated in 16 metabolic pathways, which are mainly involved in alanine metabolism, aminoacyl-tRNA biosynthesis, and glutathione metabolism. The key downregulated metabolite is glutamine, which is mainly assigned to D-glutamine and D-glutamate metabolism, purine metabolism, and pyrimidine metabolism. Results showed that Mep-induced metabolic pathways of energy, lipids, and amino acids in adipose tissue were disturbed (Figure 7).

### 3.7. Analysis of the Metabolic Function of Adipose Tissue in Rats of ND, LD, and HD Groups

Leucine, isoleucine, and valine are three common branched-chain amino acids (BCAAs) in proteins. Elevated levels of BCAAs in adipose tissue may be due to decreased expression of BCAAs catabolic enzymes [15,16]. In addition, BCAAs help to activate NADPH oxidase and produce mitochondrial reactive oxygen species (ROS), which in turn assists cellular inflammation and oxidative stress [17]. Therefore, elevated content of BCAAs may be related to the oxidative stress response induced by Mep in rats. Glutamine is helpful to tricarboxylic acid (TCA) anaplerosis, glutathione biosynthesis, and amino acid biosynthesis [18]. Decreased glutamine indicates abnormalities in the glutamate and glutamine cycle [19]. It has been shown that glutamine is involved in signal transduction, apoptosis, and autophagy of tumor cells [20]. Serine is a main methyl donor, which is necessary for the growth of cells and tissues. It has a strong effect on the catalytic activity of many enzymes [21,22,23]. Glycine is an intermediate in metabolism of serine and threonine, which is also involved in immune functions, anti-inflammatory processes, and antioxidant responses [24]. Glycine can decrease protein carbonylation and lipid peroxidation by lowering the release of superoxide radicals [25]. The glycine, serine, and threonine pathway plays a vital role in metabolic changes. It may also provide a valuable precursor for energy metabolism in TCA cycle [26]. Compared with the ND group, an increase of glycine and serine in the HD group may cause TCA cycle disorders. Proline is one of the most important amino acids for protein synthesis in the human body. Proline metabolism plays a main role in tumor development [27]. Arginine underwent hydrolysis, the formed ornithine can be changed into polyamines and proline, and the urea from its metabolism is drained by kidneys [28]. Proline was disturbed in Mep-exposed groups, and its upregulation may be related to the decrease of metabolic level [29]. Hepatocytes deal with toxic ammonia by some biochemical reactions to produce urea. The urea cycle is closely related to other metabolic pathways, indicating that abnormal expression of almost all enzymes may alter urea cycle metabolites to help tumor growth [30]. Under normal conditions, almost all urea is filtered by the kidneys. The levels of urea in adipose tissues were significantly increased in Mep-exposed groups. The reason may be that Mep provides more raw materials for the synthesis of urea, so that urea cannot be excreted in time. It may also be because Mep induced kidney damage. Creatinine is one metabolite of glycine in the body [31]. The level of creatinine is a significant indicator for studying renal function [32]. Creatinine levels were elevated in the study, indicating insufficiency of kidney function because of exposure to Mep [33].

Fatty acids affect the physiology of cells and tissues [34]. Compared with ND group, contents of glycerol, hexadecanoic acid, octadecanoic acid, arachidonic acid, and oleic acid in HD group decreased, and the level of 9, 12-octadecadienoic acid increased. Most fatty acid metabolites were significantly reduced, suggesting an increased metabolic rate of fatty acids broken down by β-oxidation. The result may be related to the enhancement of fatty acid metabolism caused by insufficient glucose metabolic energy. Stearoyl-CoA desaturase 1(SCD1) is one rate-limiting enzyme that promotes the formation of monounsaturated fatty acids [35]. We believed that the increase in 9, 12-octadecadienoic acid and the decrease in most fatty acids in Mep-exposed groups were closely related to SCD1 enzymatic activity. Excessive consumption of fat results in increased production of ROS [36]. ROS can lead to expression and secretion of inflammatory adipokines, inducing oxidative stress [37]. Arachidonic acid (AA) is the precursor of inflammatory response factors and an important second messenger in various cell signal transduction pathways [38]. When inflammatory substances invade the organism, AA is broken down into free forms and enters the cell fluid. Therefore, it is speculated that the decrease of AA content may be due to the production of inflammatory factors after Mep exposure. Changes in hexadecanoic acid contents may be related to lipid homeostasis regulation. Hexadecanoic acid has been shown to be related to insulin response [39]. In addition, hexadecanoic acid also contributes to lipoapoptosis by producing the hazardous metabolite lysophospholipid-choline [40]. In comparison with other saturated fatty acids, octadecanoic acid lowers LDL cholesterol. Octadecanoic acid inhibits growth below a specific concentration, and higher concentrations may cause cytotoxicity. Oleic acid inhibits the increase of octadecanoic acid in expression of the intercellular adhesion molecule (ICAM-1) [41]. Cholesterol is mainly formed in the liver, which is the precursor of some steroid hormones and vitamin D3. Cholesterol is indispensable for keeping life activities of cell membranes [42]. Mep-exposed groups may have lower cholesterol levels as a result of abnormal hepatic metabolism.

Pyruvate can produce lactic acid by pyruvate dehydrogenase complex [43]. The lactic acid level of adipose tissue was trending downward, which is an indication that Mep can inhibit anaerobic glycolysis. The level of D-mannitol in HD group increased significantly. D-mannitol can alter the osmotic pressure in body, but it is not utilized by the body; it is all filtered out by the glomerulus [44]. Propionic acid has function of immunosuppressive, decreasing fatty acids level and increasing insulin sensitivity [45]. Acetic acid is a vital product of fatty acid β-oxidation, which was significantly elevated in HD group rats. It increased fatty acid β-oxidation [46]. Moreover, propionate may play important physiological functions in adipogenesis by influencing G protein-coupled receptor activity [47]. Phosphoric acid is essential for bones and kidneys [48]. Imbalances in phosphate levels can bring about bone and kidney damage.

Pentanedioic acid and acetamide showed good sensitivity and specificity. In the metabolism of amino acids, pentanedioic acid is naturally generated in the body. Defects in metabolic network of pentanedioic acid result in pentanedioic aciduria, along with accumulation of toxic by-products. It may induce severe encephalopathy [49]. The massive accumulation of pentanedioic acid in Mep-exposed groups may be due to the obstacle of amino acid metabolism. Acetic acid is the source of acetamide, which primarily influences glutamate and glutamine metabolism. The acetamide content has increased in Mep-exposed groups, which is consistent with abovementioned results. In addition, acetamide can also lead to hepatoma [50].

## 4. Conclusions

To our knowledge, this is the first time that untargeted metabolomics study has been performed to investigate differential metabolites and possible toxic mechanism of Mep on adipose tissue. Twenty-six differential metabolites were screened. Levels of amino acids, such as glycine, valine, leucine, isoleucine, serine, proline, phenylalanine and alanine were higher in HD group than those in ND group. By contrast, contents of fatty acids including propanoic acid, hexadecanoic acid, octadecanoic acid, arachidonic acid, and oleic acid decreased in rats exposed to Mep. In comparison with ND group, levels of propionate, phosphoric acid, acetamide, creatinine, D-mannitol, pentanedioic acid, and acetic acid increased, while contents of lactic acid, urea, cholesterol, and glycerol decreased in the HD group. Eight major perturbed metabolic pathways were found with the exposure of Mep, which were linoleic acid metabolism, phenylalanine, tyrosine, and tryptophan biosynthesis, phenylalanine metabolism, arachidonic acid metabolism, glycine, serine, and threonine metabolism, glycerolipid metabolism, alanine, aspartate, and glutamate metabolism, and glyoxylate and dicarboxylic acid metabolism. This study lays a solid foundation for clarifying the mechanism of Mep toxicity.

## Figures and Tables

**Table 1:** Differential metabolites detected in adipose tissue of rats in the high-dose group by metabolomic analysis based on GC-MS.

Metabolite	RT (min)	*p*-Value	VIP	Fold Change (HD-ND)	FDR	Trend	Metabolic Pathways
Lactic acid	5.30	1.90 × 10^−5^	2.13	0.25	3.53 × 10^−5^	↓	Glycolysis/Gluconeogenesis
Propanoic acid	8.94	7.00 × 10^−3^	3.31	0.69	7.28 × 10^−3^	↓	Propionic acid metabolism
Alanine	10.03	1.30 × 10^−5^	1.11	1.72	2.60 × 10^−5^	↑	Alanine, aspartate, and glutamate metabolism
Glycine	10.51	9.80 × 10^−9^	1.07	3.28	3.64 × 10^−8^	↑	Glycine, serine, and threonine metabolism
Valine	13.16	4.00 × 10^−9^	1.03	3.48	1.73 × 10^−8^	↑	Valine, leucine and isoleucine biosynthesis
Urea	14.19	1.20 × 10^−8^	1.99	0.50	3.90 × 10^−8^	↓	Urea cycle
Leucine	14.71	1.50 × 10^−4^	1.90	2.05	2.17 × 10^−4^	↑	Valine, leucine and isoleucine biosynthesis
Glycerol	14.9	3.00 × 10^−3^	2.29	0.67	3.39 × 10^−3^	↓	Glycerolipid metabolism
Isoleucine	15.28	3.50 × 10^−9^	1.19	2.15	1.82 × 10^−8^	↑	Valine, leucine and isoleucine biosynthesis
Serine	17.16	5.40 × 10^−5^	1.12	1.52	8.78 × 10^−5^	↑	Glycine, serine, and threonine metabolism
Propionate	18.88	1.70 × 10^−9^	1.05	1.69	1.11 × 10^−8^	↑	Pantothenate and CoA biosynthesis
Acetic acid	20.70	1.70 × 10^−9^	1.04	1.58	2.74 × 10^−4^	↑	TCA cycle
Proline	21.07	1.40 × 10^−4^	1.70	1.52	2.14 × 10^−4^	↑	Arginine and proline metabolism
Pentanedioic acid	21.30	3.60 × 10^−10^	1.67	3.64	4.68 × 10^−9^	↑	Pentose and glucuronate interconversions
Creatinine	22.87	3.70 × 10^−8^	1.11	3.15	9.62 × 10^−8^	↑	Arginine and proline metabolism
Phenylalanine	23.49	1.10 × 10^−9^	1.16	2.44	9.53 × 10^−9^	↑	Phenylalanine, tyrosine, and tryptophan biosynthesis
Acetamide	26.14	2.30 × 10^−10^	1.82	8.90	5.98 × 10^−9^	↑	Phenylalanine metabolism
Phosphoric acid	26.82	3.30 × 10^−6^	3.27	3.29	7.15 × 10^−6^	↑	Propionic acid metabolism
Glutamine	27.14	2.30 × 10^−8^	1.01	0.30	6.64 × 10^−8^	↓	Alanine, aspartate, and glutamate metabolism
D-Mannitol	30.44	2.70 × 10^−4^	1.16	1.84	3.51 × 10^−4^	↑	Fructose and mannose metabolism
Hexadecanoic acid	32.01	7.40 × 10^−4^	4.06	0.61	8.75 × 10^−4^	↓	Fatty acid biosynthesis
9,12-Octadecadienoic acid	35.16	9.70 × 10^−8^	3.28	2.20	2.29 × 10^−7^	↑	Fatty acid biosynthesis
Octadecanoic acid	35.48	3.30 × 10^−4^	1.79	0.62	4.09 × 10^−4^	↓	Fatty acid biosynthesis
Arachidonic acid	39.52	1.20 × 10^−2^	1.31	0.77	1.20 × 10^−2^	↓	Arachidonic acid metabolism
Oleic acid	41.94	2.80 × 10^−5^	1.93	0.46	4.85 × 10^−5^	↓	Fatty acid biosynthesis
Cholesterol	44.48	5.00 × 10^−3^	1.12	0.71	5.42 × 10^−3^	↓	Steroid hormone biosynthesis

“↑” represents an increase in content of high-dose group compared to normal group; “↓” represents a decrease in high dose group compared to normal group.

**Table 2:** Pathway analysis result with MetaboAnalyst 5.0.

	Total	Expected	Hits	Raw *p*	−log(*p*)	Holm *p*	FDR
Glyoxylate and dicarboxylate metabolism	32	0.51613	3	0.013508	1.8694	1	0.28367
Phenylalanine, tyrosine, and tryptophan biosynthesis	4	0.064516	1	0.063032	1.2004	1	0.59703
Alanine, aspartate, and glutamate metabolism	28	0.45161	2	0.073113	1.136	1	0.59703
Linoleic acid metabolism	5	0.080645	1	0.078183	1.1069	1	0.59703
Phenylalanine metabolism	10	0.16129	1	0.15048	0.82252	1	0.90288
Glycerolipid metabolism	16	0.25806	1	0.23006	0.63815	1	1
Glycine, serine, and threonine metabolism	33	0.53226	1	0.41857	0.37823	1	1
Arachidonic acid metabolism	36	0.58065	1	0.44686	0.34983	1	1

## Data Availability

The data supporting the results of the study are included in the article.

## Supplementary Materials

The following supporting information can be downloaded at: https://www.mdpi.com/article/10.3390/foods12040867/s1, Figure S1: Changes in body weight of rats in ND, LD, and HD groups. Figure S2: Linoleic acid metabolic pathway in rats among different groups. Table S1. ROC curve and its statistical parameters. Table S2. Metabolic pathways list.
