# Supplementary material for: Untargeted Metabolite Profiling of Adipose Tissue in Rats Exposed to Mepiquat
Source: Foods. 2023 Feb 17;12(4):867. doi: 10.3390/foods12040867 (PMC9956293; doi:10.3390/foods12040867)
Supplement: Supplementary file 1 [file foods-12-00867-s001.zip › foods-2164088-supplementary.pdf]

# Supplementary Materials

**Chuanqin Hu, Xinyu Song, Zhenzhen Shao, Yingli Liu, Jing Wang * and Baoguo Sun**

China-Canada Joint Lab of Food Nutrition and Health (Beijing), Beijing Advanced Innovation Center for Food Nutrition and Human Health, Beijing Engineering and Technology Research Center of Food Additives, Beijing Laboratory for Food Quality and Safety, Beijing Technology and Business University (BTBU), 11 Fucheng Road, Beijing 100048, China

* Correspondence: wangjing@th.btbu.edu.cn

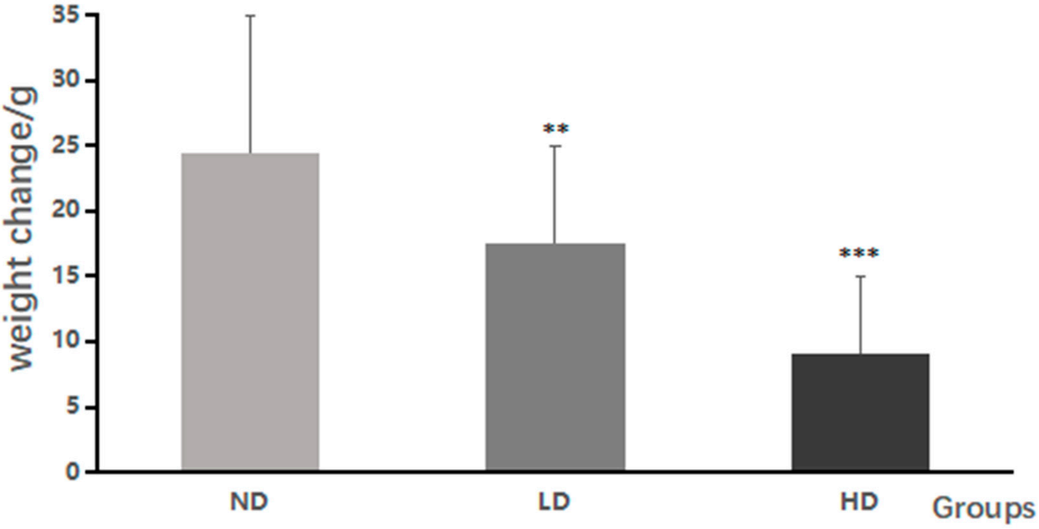


**Figure S1.** Changes in body weight of rats in ND, LD and HD groups ("*" represents $p<0.05$, "**" represents $p<0.01$, and "***" represents $p<0.001$, ND group: Normal Diet group, n=10; LD group : Low-dose Diet group, n=10; HD group: High-dose Diet group, n=10)

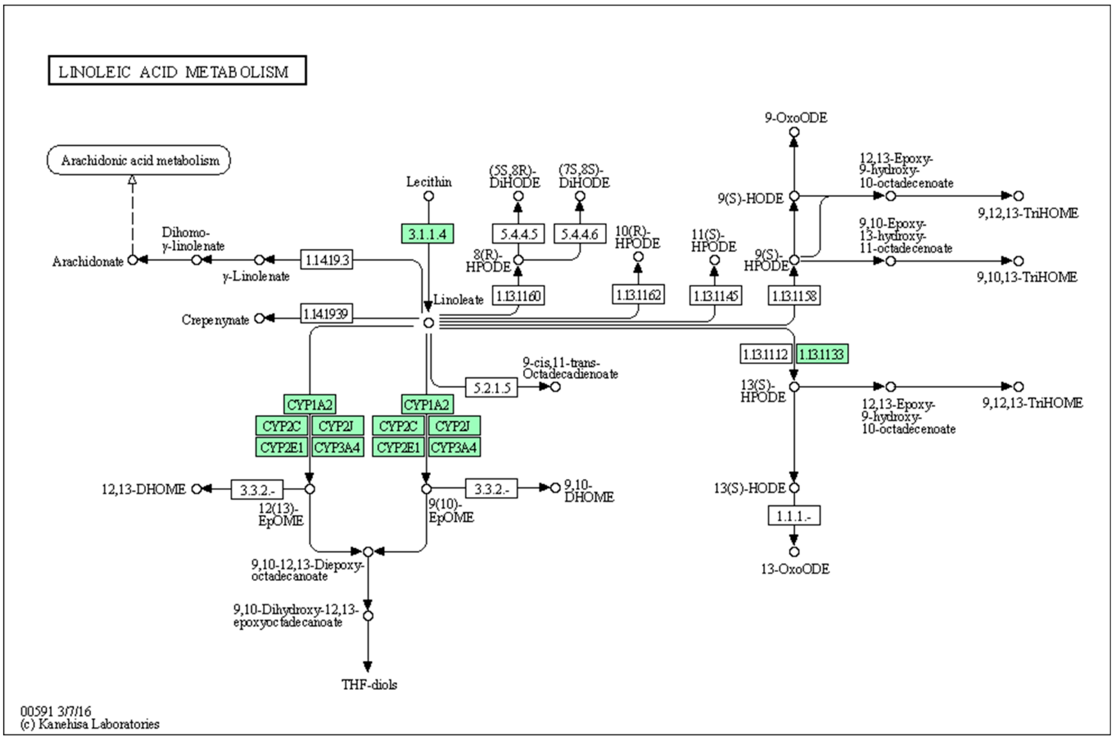


**Figure S2.** Linoleic acid metabolic pathway in rats among different groups.

**Table S1.** Areas under ROC curve (AUC) and the statistic significances.

| Metabolite | AUC | Standard error | Sig. |
|---|---|---|---|
| Lactic acid | 0.978 | 0.028 | 0 |
| Propanoic acid | 0.878 | 0.091 | 0.006 |
| Alanine | 0.878 | 0.085 | 0.006 |
| Glycine | 0.856 | 0.100 | 0.009 |
| Valine | 0.878 | 0.097 | 0.006 |
| Urea | 0.989 | 0.018 | 0 |
| Leucine | 0.889 | 0.095 | 0.004 |
| Glycerol | 0.900 | 0.087 | 0.003 |
| Isoleucine | 0.967 | 0.035 | 0.001 |
| Serine | 0.967 | 0.038 | 0.001 |
| Propionate | 0.900 | 0.070 | 0.003 |
| Acetic acid | 0.900 | 0.086 | 0.003 |
| Proline | 0.867 | 0.085 | 0.007 |
| Pentanedioic acid | 0.944 | 0.051 | 0.001 |
| Creatinine | 0.911 | 0.072 | 0.003 |
| Phenylalanine | 0.889 | 0.083 | 0.004 |
| Acetamide | 0.956 | 0.043 | 0.001 |
| Phosphoric acid | 0.889 | 0.095 | 0.004 |
| Glutamine | 0.956 | 0.048 | 0.001 |
| D-Mannitol | 0.833 | 0.109 | 0.014 |
| Hexadecanoic acid | 0.944 | 0.052 | 0.001 |
| 9,12-Octadecadienoic acid | 0.978 | 0.028 | 0 |
| Octadecanoic acid | 0.989 | 0.018 | 0 |
| Arachidonic acid | 0.856 | 0.088 | 0.009 |
| Oleic acid | 0.989 | 0.018 | 0 |
| Cholesterol | 0.878 | 0.078 | 0.006 |

**Table S2.** Metabolic pathways list

| Metabolic pathway ID | Metabolism | Metabolism ID | Metabolic pathway |
|---|---|---|---|
| A | Carbohydrate metabolism | 1 | Pyruvate metabolism |
| A | Carbohydrate metabolism | 2 | Glycolysis / Gluconeogenesis |
| A | Carbohydrate metabolism | 3 | Glyoxylate and dicarboxylate metabolism |
| A | Carbohydrate metabolism | 4 | Propanoate metabolism |
| A | Carbohydrate metabolism | 5 | Galactose metabolism |
| A | Carbohydrate metabolism | 6 | Pentose and glucuronate interconversions |
| A | Carbohydrate metabolism | 7 | Fructose and mannose metabolism |
| B | Lipid metabolism | 8 | Biosynthesis of unsaturated fatty acids |
| B | Lipid metabolism | 9 | Linoleic acid metabolism |
| B | Lipid metabolism | 10 | Primary bile acid biosynthesis |
| B | Lipid metabolism | 11 | Glycerolipid metabolism |
| B | Lipid metabolism | 12 | Arachidonic acid metabolism |
| B | Lipid metabolism | 13 | Fatty acid elongation |
| B | Lipid metabolism | 14 | Fatty acid degradation |
| B | Lipid metabolism | 15 | Steroid biosynthesis |
| B | Lipid metabolism | 16 | Fatty acid biosynthesis |
| B | Lipid metabolism | 17 | Steroid hormone biosynthesis |
| C | Amino acid metabolism | 18 | Valine, leucine and isoleucine biosynthesis |
| C | Amino acid metabolism | 19 | Valine, leucine and isoleucine degradation |
| C | Amino acid metabolism | 20 | Phenylalanine, tyrosine and tryptophan biosynthesis |
| C | Amino acid metabolism | 21 | Phenylalanine metabolism |
| C | Amino acid metabolism | 22 | Arginine biosynthesis |
| C | Amino acid metabolism | 23 | Alanine, aspartate and glutamate metabolism |
| C | Amino acid metabolism | 24 | Glycine, serine and threonine metabolism |
| C | Amino acid metabolism | 25 | Arginine and proline metabolism |
| D | Metabolism of other amino acids | 26 | Selenocompound metabolism |
| D | Metabolism of other amino acids | 27 | Glutathione metabolism |
| E | Metabolism of cofactors and vitamins | 28 | Pantothenate and CoA biosynthesis |
| E | Metabolism of cofactors and vitamins | 29 | Porphyrin and chlorophyll metabolism |
| F | Other metabolism | 30 | Aminoacyl-tRNA biosynthesis |

**Table S2.** Continued

| Metabolic pathway ID | Metabolism | Metabolism ID | Metabolic pathway |
|---|---|---|---|
| F | Other metabolism | 31 | Purine metabolism |
| F | Other metabolism | 32 | Pyrimidine metabolism |
| F | Other metabolism | 33 | D-Glutamine and D-glutamate metabolism |
| F | Other metabolism | 34 | Nitrogen metabolism |
